# Outcome of preventive nursing intervention, prophylactic anticoagulation and the use of the Caprini score on venous thromboembolism after varicose vein surgery

**DOI:** 10.4314/ahs.v23i3.72

**Published:** 2023-09

**Authors:** Ting Zheng, Xiaofei Zheng

**Affiliations:** 1 Cardiothoracic Vascular Surgery, Kecheng District People's Hospital, Zhejiang Province 324300; 2 Quality Management Section, Kecheng District People's Hospital, Zhejiang Province 324300

**Keywords:** Varicose vein surgery, nursing intervention, Caprini score

## Abstract

**Objective:**

This research was devoted to estimate the outcome of preventive nursing intervention on venous thromboembolism (VTE) after varicose vein surgery in lower extremities.

**Methods:**

A total of 516 subjects with varicose veins of lower extremities (from January 2020 to January 2022) treated in our hospital were separated into observation subgroup (n = 258) and control subgroup (n = 258) at random. The conventional nursing intervention was applied in the control subgroup, preventive nursing intervention, prophylactic anticoagulation and the use of Caprini score was applied in the observation subgroup. The levels of blood indexes [hemoglobin (Hb), platelet (PLT) and D-dimer (D-D)] were compared between the two subgroups before operation and 7 days postoperative, the occurrence of subcutaneous congestion, lower limb swelling and pain, VTE and nursing satisfaction of the subject 4 (four) weeks after discharge.

**Results:**

After intervention, the levels of PLT and D-D in the observation subgroup were notably lower than those in the control subgroup. Four weeks after discharge, the incidence of subcutaneous congestion, lower limb swelling and pain, there had no notable difference in two subgroups Versus the control subgroup, VTE in the observation subgroup was notably lower and nursing satisfaction was higher.

**Conclusion:**

Preventive nursing intervention can reduce the level of PLT and D-D, restrain in the incidence of VTE and improve the nursing satisfaction of subjects with varicose veins of lower extremities after varicose vein surgery of lower limb.

## Background

Varicose veins of lower extremities are common vascular surgical diseases in clinic. According to epidemiological statistics, the incidence of varicose veins of lower extremities is about 15% in adult males and about 25% in adult females 1. The clinical manifestation of the disease is presented as lumbricoid in the lower limbs, which often twists and turns into clumps, which is more obvious when standing, and raised part can be alleviated when raising the limbs. If the disease is further aggravated, it will affect the skin nutrition supply of the lower limbs. There is local itching, pain, swelling symptoms 2-4.

Surgery is an effective method for the treatment of varicose veins of lower extremities, but venous thromboembolism (VTE) is easy to occur postoperative, which not only increases the difficulty of treatment but also increases the risk of death 5,6. Vascular surgeons attach great importance on how to take effective measures to prevent the occurrence of VTE and improve the prognosis of subjects with varicose veins of lower extremities. In this study, preventive nursing intervention was applied to postoperative subjects with varicose veins of lower extremities in order to explore its effect in preventing the occurrence of venous thromboembolism. It is reported as follows.

## Methods

### Design and procedures

This was a randomized controlled double-arm trial with repeated measurements from January 2020 to January 2022. In this randomized controlled trial, the conventional nursing intervention group was compared with preventive nursing intervention, prophylactic anticoagulation and the use of the Caprini score group in order to expect a significant improvement in the levels of blood indexes [hemoglobin (Hb), platelet (PLT) and D-dimer (D-D)], the occurrence of subcutaneous congestion, lower limb swelling and pain, VTE and nursing satisfaction at two follow-up points. Two assessments were conducted: At baseline and 7 days postoperative, the control subgroup received conventional nursing intervention. The observation subgroup was preventive nursing intervention, prophylactic anticoagulation and the use of the Caprini score group, which was based on conventional nursing intervention, adopted preventive nursing intervention, prophylactic anticoagulation and used the Caprini score.

### Setting and Participants

The eligibility and exclusion criteria of subjects were evaluated according to the inclusion situation. After screening, the participants were randomly assigned.

**Inclusion criteria:**

1. All subjects were diagnosed as varicose veins of lower limbs.
2. All subjects underwent lower limbs varicose surgery.
3. The age of all subjects ≥ 18 years.
4. The subjects were informed of the study.

**Exclusion criteria:**

1. Accompanied by coagulation dysfunction of the subjects.
2. The subjects with recent history of acute bleeding.
3. The subjects with severe cardiovascular and cerebrovascular diseases.
4. There was venous thrombosis before operation. It was sent to the control subgroup or the observation subgroup through a random number table. The subjects were not dropped out.

The ethics committee of the hospital approved this study

## Procedure

### Nursing methods

The subjects of the control subgroup were taken routine nursing intervention. Before the operation, the subjects and their families were informed of the relevant procedures and matters needing attention, and the questions of the subjects and their families were answered. The nursing during the operation was done well. The subjects' vital signs were monitored after the operation, the subject's condition was observed, the subjects and their families were given health education, the subjects were told how to use the medicine, and the subjects' families were instructed to give the subjects reasonable diet.

The subjects of the observation subgroup were applied, preventive nursing intervention and Caprini scale to assess the risk of VTE. 0 points was represented very low risk, 1 to 2 points was represented low risk, 3 to 4 points was represented moderate risk, and ≥ 6 points was represented high risk. The corresponding preventive measures were given according to different risk levels. Low risk patients were advised to exercise early and given physical prevention. Patients with moderate risk were given physical prevention and drug prevention for 7-10 days. The highly dangerous patients were given physical prevention and drug prevention for 11-35 days. For drug prevention, the bleeding risk of patients' needs to be assessed. For patients with high bleeding risk, drug prevention should be used cautiously.

### Physical prevention

1. Intravenous infusion of lower limbs was strictly prohibited. The affected limb was raised to make it 20cm higher than the heart plane. Patients were given ankle pump exercise immediately after the recovery of limb consciousness after operation, and got out of bed for 24 hours after operation. After the limb regains consciousness, ankle pump movement was carried out immediately.

2. If the subjects' condition was allowed, the subjects were supervised to drink more than 2500mL of water every day. After the suture was removed, elastic stockings of appropriate size were selected, the subjects and their family members were guided to wear them correctly to prevent congestion of the venous system.

### Drug prevention

At 6h and 12h postoperative, 4000U of low molecular weight heparin sodium (Qilu Pharmaceutical Factory, National Drug Approval No. H200000) was injected subcutaneously twice a day for 5 consecutive days. Falling or bumping during medication, and whether there was bleeding tendency was monitored during brushing teeth. The dose of low molecular weight heparin sodium was adjusted according to the activity of anti-Xa factor. After 3 days of treatment with low molecular weight heparin, warfarin was taken orally, and the dosage was adjusted according to the international standardized ratio (INR) to keep the INR between 2.0 and 3.0.

### The scores of Caprini scale

Low risk 17 cases (6.59%), moderate risk 205 cases (79.46%), severe risk 36 cases (13.95%). Among them, 211 patients received prophylactic anticoagulation therapy.

### Observations

1. **Blood index detection:**

The levels of hemoglobin (Hb), platelet (PLT) and D-dimer (D-D) were detected before and 7 days after the operation.

2. 4 weeks after discharge, the subcutaneous congestion, lower limb swelling, pain and VTE of the subjects were monitored by telephone follow-up or outpatient. Subcutaneous congestion was characterized by local skin cyanosis or protuberance, and lower limb swelling and pain were characterized by local high skin temperature, pain, and increased tension. VTE was diagnosed by color ultrasound, the whole lower limb was scanned.

3. The subjects' nursing satisfaction was evaluated, and issued self-made satisfaction questionnaire, which was divided into three options: Satisfactory, general satisfactory and dissatisfied. Total satisfaction= (satisfied cases + general cases)/total cases × 100%.

### Statistical methods

The data in this paper were analysed by SPSS 21.0 statistical software. The subject's sex, side, VET incidence and other count data were expressed by the rate (%). The chi square test was applied for comparison between subgroups. The subjects' blood indicators were the measurement data, expressed by mean ± standard deviation (^-^x±s), it conformed to normal distribution and had uniform variances. Independent sample t-test was applied for inter _subgroup comparison and paired sample t-test was applied for intra subgroup comparison. The difference was statistically notable if *P*<0.05.

## Results

### General data

In the observation subgroup, 113 males and 145 females were contained, the average age was (60.18 ± 6.39) years, containing 214 cases of varicose great saphenous vein, 44 cases of varicose small saphenous vein, 160 cases of unilateral and 98 cases of bilateral. 55 cases underwent high ligation and stripping of the great saphenous vein, and 97 cases underwent resection of the great saphenous vein, 106 cases underwent microwave ablation were concluded. In the control subgroup, 118 males and 140 females were contained, the average age was (61.05 ± 6.72) years. 219 cases of great saphenous varices, 39 cases of small saphenous varices, 164 cases of unilateral and 94 cases of bilateral were maintained. 50 cases underwent high li-gation and stripping of great saphenous vein, 100 cases underwent resection of great saphenous vein, and 108 patients underwent microwave ablation were concluded. The baseline data of the two subgroups were similar (*P*>0.05) and comparable.

### Comparison of blood indexes

Before the intervention, there was no notable difference in blood indicators between the two subgroups (*P*>0.05). After the intervention, the levels of PLT and D-D in the two subgroups increased, but the levels of PLT and D-D in the observation subgroup were notably lower than those in the control subgroup (*P*<0.05). As corroborated in Table 1.

**Table 1:** Comparison of blood indicators between the two subgroups (x ± s)

Grouping	n	Hb (g/L)		PLT (10^9^/L)		D-D (mg/mL)	

		Before intervention	After intervention	Before intervention	After intervention	Before intervention	After intervention
The observation subgroup	258	124.38±22.43	97.81±11.23*	216.32±41.58	225.47±55.23*	228.37±56.68	420.18±95.65*
The control subgroup	258	124.10±21.65	98.74±12.38*	214.59±49.47	238.78±56.18*	225.46±57.42	458.27±115.52*
*t*		0.144	0.894	0.430	2.714	0.579	4.079
*P*		0.885	0.372	0.667	0.007	0.563	0.001

Note: Compared with the same subgroup before intervention

* *P* < 0.05.

### Comparison of follow-up between the two subgroups

There was no notable difference between the two subgroups in the incidence of subcutaneous congestion, lower limb swelling and pain (*P*>0.05). In the observation subgroup, 1 case had VTE, which was located in the femoral vein of the affected limb; There were 7 cases of VTE in the control subgroup, including 3 cases of shanks intermuscular vein, 2 cases of femoral vein, 1 case of popliteal vein, 1 case of iliac vein and femoral vein. The VTE in the observation subgroup was lower than that in the control subgroup, with a notable difference (*P*<0.05). As corroborated in Table 2.

**Table 2:** Comparison of follow-up [n (%)]

Grouping	n	Subcutaneous congestion	Lower limb swelling and pain	VTE
The observation subgroup	258	5 (1.94)	16 (6.20)	1 (0.39)
The control subgroup	258	9 (3.49)	20 (7.75)	7 (2.71)
*χ^2^*		1.175	0.478	4.571
*P*		0.278	0.489	0.033

### Comparison of nursing satisfaction between the two subgroups

1 case of VET in the observation subgroup was satisfied, while in the control subgroup, 1 case was satisfied, 2 cases were average and 4 cases were not satisfied. The nursing satisfaction of the observation subgroup was higher than that of the control subgroup, with a statistically notable difference (*P*<0.05), as corroborated in Table 3.

**Table 3:** Comparison of nursing satisfaction [n (%)]

Grouping	n	Satisfactory	General satisfactory	Dissatisfied	Total satisfaction
The observation subgroup	258	165 (63.95)	76 (29.46)	17 (6.59)	241 (93.41)
The control subgroup	258	121 (46.90)	105 (40.70)	32 (12.40)	226 (87.60)
*χ^2^*					5.074
*P*					0.024

## Discussion

VTE includes deep vein thrombosis and pulmonary thromboembolism 7. Deep vein thrombosis refers to a disease in which blood clots in deep veins, leading to vascular obstruction and venous reflux disorder. Pulmonary thromboembolism refers to a disease in which the venous system or right heart blood thrombus falls off, eventually causing pulmonary artery obstruction and affecting respiratory function and pulmonary circulation 8-10. VTE has a high incidence rate, which is the third largest vascular disease after coronary syndrome and stroke. In addition, VTE also has the characteristics of high mortality, high misdiagnosis rate and high missed diagnosis rate, often occur after surgery 11,12. It has been reported that the incidence of VTE in surgical subject's accounts for about 27% of the total number of VTE, but it can be prevented through reasonable intervention measures 13.

VTE is a complication of varicose veins of lower extremities. Due to the influence of anesthesia, pain, reduced exercise and other factors, the blood flow velocity of subjects with varicose veins of lower extremities decrease and the level of coagulation factors increase postoperative, providing favourable conditions for thrombosis 14-17. Preventive nursing refers that nurses predict nursing risks in advance after comprehensive analysis and judgment of subjects, and then take effective measures to intervene to avoid complications, so as to improve nursing quality and satisfaction 18,19. For subjects with varicose veins of lower extremities, that is, before the occurrence of VTE, through comprehensive evaluation, targeted nursing intervention was given to reduce the risk of VTE.

In this study, Caprini scale was applied as a risk assessment tool, which included 39 high risk factors, such as age, operation time, malignant tumor, VTE history and so on. subjects were divided into 4 risk grades according to all risk factor scores, and different intervention measures were given according to risk grades. After varicose vein surgery of lower extremities, due to the slowing down of blood flow speed, the subject's activity is reduced, the venous return is slowed down, and the body is in a hyper-coagulable state, which is manifested by the increase of PLT and D-D levels. If the hypercoagulable state is not improved in time, VTE is very likely to occur.

The results of this study showed that after the intervention, the levels of PLT and D-D in the two subgroups were higher than those before the intervention, but the levels of PLT and D-D in the observation subgroup were declined than those in the control subgroup on average, and the incidence of VTE was notably declined than that in the control subgroup, suggesting that preventive intervention could avoid excessive elevation of PLT and D-D levels, improve the postoperative hypercoagulable state of the body, and thus reduce the incidence of VTE. Analysing the reasons, the preventive intervention in this study mainly includes physical intervention and drug intervention. Physical intervention is mainly achieved through ankle pump exercise and wearing elastic socks.

Ankle pump exercise can promote blood pressure circulation and lymphatic circulation of the lower limbs of bedridden subjects, eliminate swelling, and also enhance muscle strength to avoid muscle atrophy 20,21, wearing elastic stockings can stimulate lower limb blood vessels, restore the function of damaged endothelial cells, reduce the activity of coagulation factors, and enhance the thrombolytic capacity of the body 22-24. Drug intervention is to give subjects low molecular weight heparin sodium, which can play an anticoagulant role by activating antithrombin. Some studies show that low molecular weight heparin sodium is the same as aspirin, can reduce the incidence of venous thrombosis, and can be applied to prevent the occurrence of VTE after surgery 25.

To sum it up, in the process of preventive nursing intervention, medical staff carried out early assessment and VTE risk screening for subjects, and targeted effective measures. Compared to conventional nursing, preventive nursing achieved early attention and targeted prevention, making the subjects receiving care feel better, and the incidence of VTE after surgery is low. Therefore, the subject satisfaction of the preventive nursing subgroup is higher, which is conducive to the establishment of the doctor-subject relationship.

## Figures and Tables

**Figure 1:**
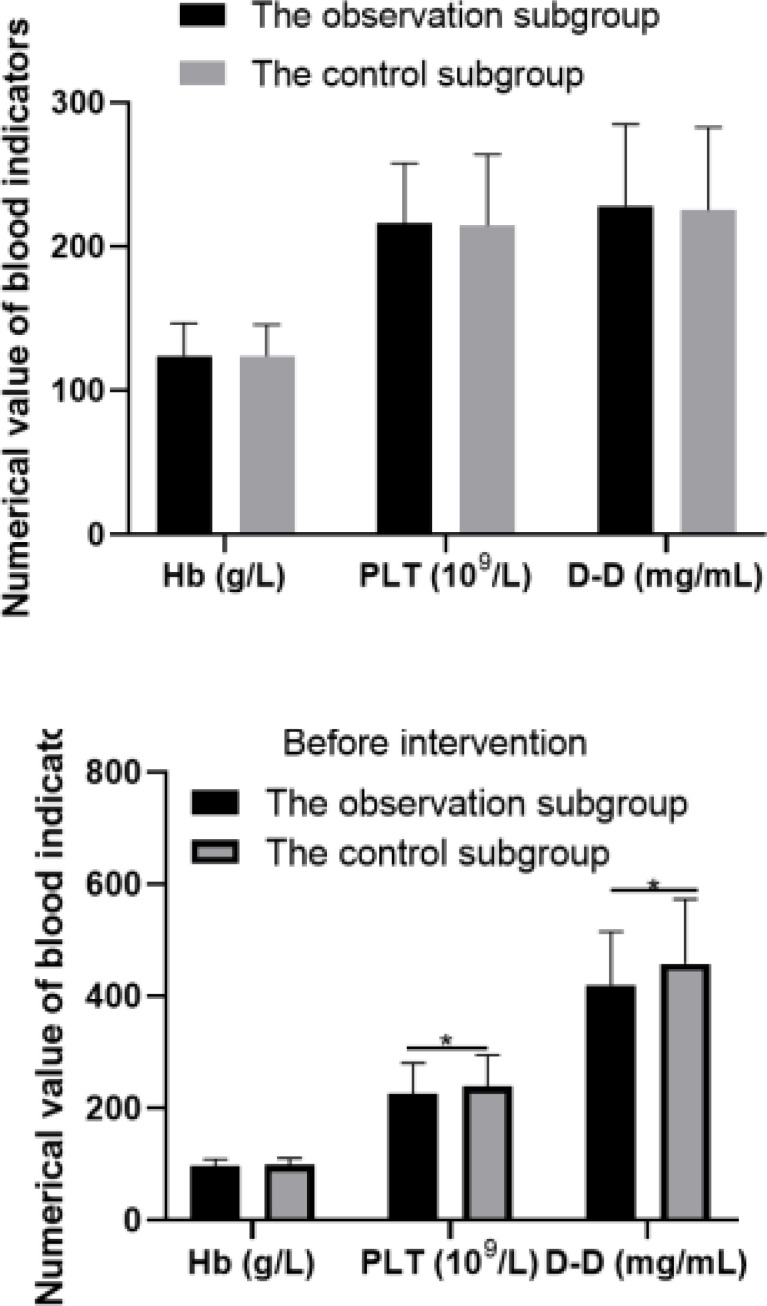
Comparison of blood indicators. * *P* < 0.05

**Figure 2:**
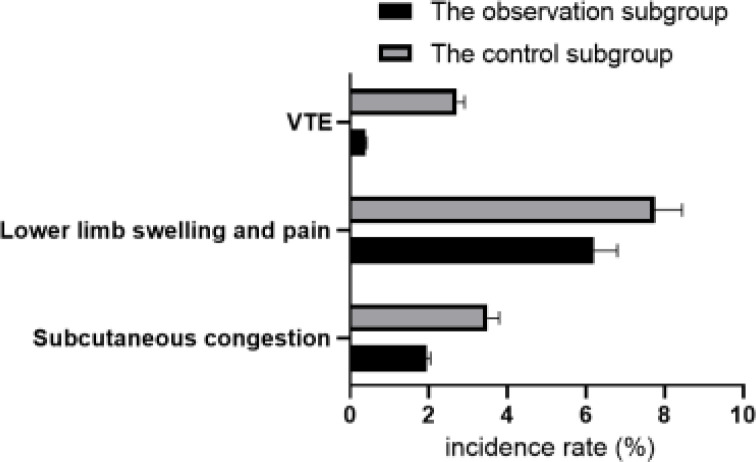
Comparison of follow-up between the two subgroups. *P<0.05

**Figure 3:**
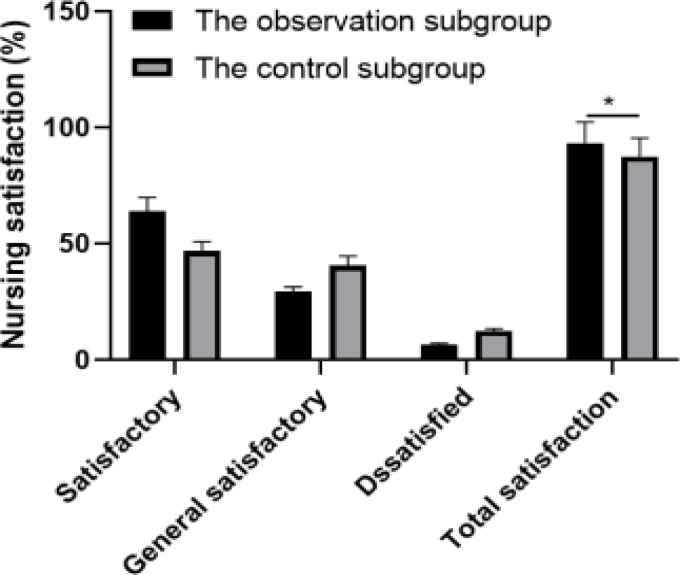
Comparison of nursing satisfaction. *P<0.05

## References

[1] Youn YJ, Lee J (2019). Chronic venous insufficiency and varicose veins of the lower extremities[J]. Korean J Intern Med.

[2] Raetz J, Wilson M, Collins K (2019). Varicose Veins: Diagnosis and Treatment[J]. Am Fam Physician.

[3] Dwyer HC, Baranowski DC, Mayer PV, Gabriele S (2018). LivRelief varicose veins cream in the treatment of chronic venous insufficiency of the lower limbs: A 6-week single arm pilot study[J]. PLoS One.

[4] Jiang H, Qiu LL, Li YY, Gu LP, Liu QG (2020). Efficacy and mechanism of fire needling bloodletting for lower extremity varicose veins[J]. Zhongguo Zhen Jiu.

[5] Luo LH, Chen Z, Hu LN, Ma C, Xiao EH (2019). Tumescence Anesthesia Solution-Assisted Laser Ablation Treatment of Lower Limb Varicose Veins: The Effect of Temperature of the Tumescence Anesthesia Solution on Intraoperative and Postoperative Pain, Clinical Observations, and Comprehensive Nursing Care[J]. J Perianesth Nurs.

[6] Kappa-Markovi K, Jalaie H, Özhan-Hasan H, Deges M, Rass K (2021). Intermittent pneumatic compression after varicose vein surgery[J]. J Vasc Surg Venous Lymphat Disord.

[7] Kundal A, Kumar N, Rajput D, Chauhan U (2020). Great saphenous vein sparing versus stripping in Trendelenburg operation for primary varicose veins: a prospective study[J]. Pol Przegl Chir.

[8] Vedantham S, Goldhaber SZ, Julian JA, Kahn SR, Jaff MR, Cohen DJ, Magnuson E, Razavi MK, Comerota AJ, Gornik HL, Murphy TP, Lewis L, Duncan JR, Nieters P, Derfler MC, Filion M, Gu CS, Kee S, Schneider J, Saad N, Blinder M, Moll S, Sacks D, Lin J, Rundback J, Garcia M, Razdan R, VanderWoude E, Marques V, Kearon C, ATTRACT Trial Investigators (2017). Pharmacomechanical Catheter-Directed Thrombolysis for Deep-Vein Throm-bosis[J]. N Engl J Med.

[9] Farhan A, Bukhari M, Umar J, Raza MA (2019). Oral Rivar-oxaban in Symptomatic Deep Vein Thrombosis[J]. J Coll Physicians Surg Pak.

[10] Freund Y, Chauvin A, Jimenez S, Philippon AL, Curac S, Fémy F, Gorlicki J, Chouihed T, Goulet H, Montassier E, Dumont M, Lozano Polo L, Le Borgne P, Khellaf M, Bouzid D, Raynal PA, Abdessaied N, Laribi S, Guenezan J, Ganansia O, Bloom B, Miró O, Cachanado M, Simon T (2021). Effect of a Diagnostic Strategy Using an Elevated and Age-Adjusted D-Dimer Threshold on Thromboembolic Events in Emergency Department Patients with Suspected Pulmonary Embolism: A Randomized Clinical Trial[J]. JAMA.

[11] Wallace T, El-Sheikha J, Nandhra S, Leung C, Mohamed A, Harwood A, Smith G, Carradice D, Chetter I (2018). Long-term outcomes of endovenous laser ablation and conventional surgery for great saphenous varicose veins[J]. Br J Surg.

[12] Sandhya PA, Mohil RS, Sricharan R (2020). Randomised controlled study to compare radiofrequency ablation with minimally invasive ultrasound-guided non-flush ligation and stripping of great saphenous vein in the treatment of varicose veins[J]. Ann R Coll Surg Engl.

[13] Guntupalli SR, Brennecke A, Behbakht K, Tayebnejad A, Breed CA, Babayan LM, Cheng G, Ramzan AA, Wheeler LJ, Corr BR, Lefkowits C, Sheeder J, Matsuo K, Flink D (2020). Safety and Efficacy of Apixaban vs Enoxaparin for Preventing Postoperative Venous Thromboembolism in Women Undergoing Surgery for Gynecologic Malignant Neoplasm: A Randomized Clinical Trial[J]. JAMA Netw Open.

[14] Weitz JI, Bauersachs R, Becker B, Berkowitz SD, Freitas MCS, Lassen MR, Metzig C, Raskob GE (2020). Effect of Osocimab in Preventing Venous Thromboembolism Among Patients Undergoing Knee Arthroplasty: The FOXTROT Randomized Clinical Trial[J]. JAMA.

[15] Giustozzi M, Connors JM, Ruperez Blanco AB, Szmit S, Falvo N, Cohen AT, Huisman M, Bauersachs R, Den-tali F, Becattini C, Agnelli G (2021). Clinical characteristics and outcomes of incidental venous thromboembolism in cancer patients: Insights from the Caravaggio study[J]. J Thromb Haemost.

[16] Pannucci CJ, Fleming KI, Bertolaccini C, Moulton L, Stringham J, Barnett S, Lin J, Varghese TK (2020). Fixed or Weight-Tiered Enoxaparin After Thoracic Surgery for Venous Thromboembolism Prevention[J]. Ann Thorac Surg.

[17] Yu R, Nansubuga F, Yang J, Ding W, Li K, Weng D, Wu P, Chen G, Ma D, Wei J (2020). Efficiency and safety evaluation of prophylaxes for venous thrombosis after gynecological surgery[J]. Medicine (Baltimore).

[18] Chen Yingying, Jiang Xiaxzhou (2023). Research on early painless rehabilitation nursing intervention for patients undergoing knee arthroplasty based on evidence-based medicine theory[J]. International Journal of General Practice.

[19] Taksler GB, Hu B, DeGrandis F, Montori VM, Fagerlin A, Nagykaldi Z, Rothberg MB (2021). Effect of Individualized Preventive Care Recommendations vs Usual Care on Patient Interest and Use of Recommendations: A Pilot Randomized Clinical Trial[J]. JAMA Netw Open.

[20] Sakai K, Takahira N, Tsuda K, Akamine A (2021). Effects of intermittent pneumatic compression on femoral vein peak venous velocity during active ankle exercise[J]. J Orthop Surg (Hong Kong).

[21] Li H, Zhang W, Lu Q, Wang J, Zhi Y, Zhang L, Zhou L (2022). Which Frequency of Ankle Pump Exercise Should be Chosen for the Prophylaxis of Deep Vein Thrombosis? [J]. Inquiry.

[22] Kakkos SK, Timpilis M, Patrinos P, Nikolakopoulos KM, Papageorgopoulou CP, Kouri AK, Ntouvas I, Papa-doulas SI, Lampropoulos GC, Tsolakis IA (2018). Acute effects of graduated elastic compression stockings in patients with symptomatic varicose Veins: A randomised double Blind Placebo Controlled Trial[J]. Eur J Vasc Endovasc Surg.

[23] Shalhoub J, Lawton R, Hudson J, Baker C, Bradbury A, Dhillon K, Everington T, Gohel MS, Hamady Z, Hunt BJ, Stansby G, Warwick D, Norrie J, Davies AH (2020). Compression stockings in addition to low-molecular-weight heparin to prevent venous thromboembolism in surgical impatients requiring pharmacoprophylaxis: the GAPS non-inferiority RCT[J]. Health Technol Assess.

[24] Yang X, Zhang X, Yin M, Wang R, Lu X, Ye K (2022). Elastic compression stockings to prevent post-thrombotic syndrome in proximal deep venous thrombosis patients without thrombus removal[J]. J Vasc Surg Venous Lymphat Disord.

[25] Haac BE, O'Hara NN, Manson TT, Slobogean GP, Castillo RC, O'Toole RV, Stein DM, ADAPT Investigators (2020). Aspirin versus low-molecular-weight heparin for venous thromboembolism prophylaxis in orthopaedic trauma patients: A patient-centered randomized controlled trial[J]. PLoS One.

